# Focused attention, open monitoring and loving kindness meditation: effects on attention, conflict monitoring, and creativity – A review

**DOI:** 10.3389/fpsyg.2014.01083

**Published:** 2014-09-23

**Authors:** Dominique P. Lippelt, Bernhard Hommel, Lorenza S. Colzato

**Affiliations:** Cognitive Psychology Unit, Institute for Psychological Research and Leiden Institute for Brain and Cognition, Leiden UniversityLeiden, Netherlands

**Keywords:** open monitoring, focused attention, attention, ACC, conflict monitoring, vipassana

## Abstract

Meditation is becoming increasingly popular as a topic for scientific research and theories on meditation are becoming ever more specific. We distinguish between what is called *focused Attention meditation*, *open Monitoring meditation*, and *loving kindness* (or compassion) *meditation*. Research suggests that these meditations have differential, dissociable effects on a wide range of cognitive (control) processes, such as attentional selection, conflict monitoring, divergent, and convergent thinking. Although research on exactly how the various meditations operate on these processes is still missing, different kinds of meditations are associated with different neural structures and different patterns of electroencephalographic activity. In this review we discuss recent findings on meditation and suggest how the different meditations may affect cognitive processes, and we give suggestions for directions of future research.

## INTRODUCTION

Even though numerous studies have shown meditation to have significant effects on various affective and cognitive processes, many still view meditation as a technique primarily intended for relaxation and stress reduction. While meditation does seem to reduce stress and to induce a relaxing state of mind, it can also have significant effects on how people perceive and process the world around them and alter the way they regulate attention and emotion. Lutz et al. (2008) proposed that the kind of effect meditation has is likely to differ according to the kind of meditation that is practiced. Currently the most researched types of meditation include focused attention meditation (FAM), open monitoring meditation (OMM), and loving-kindness meditation (LKM). Unfortunately, however, the methodological diversity across the available studies with regard to sample characteristics, tasks used, and experimental design (within vs. between group; with vs. without control condition) renders the comparison between them difficult. This review is primarily focused on FAM and OMM studies^1^ and on how these two (proto-)types of meditation are associated with different neural underpinnings and differential effects on attentional control, conflict monitoring, and creativity.

## MEDITATION TYPES

Usually, FAM is the starting point for any novice meditator (Lutz et al., 2008; Vago and Silbersweig, 2012). During FAM the practitioner is required to focus attention on a chosen object or event, such as breathing or a candle flame. To maintain this focus, the practitioner has to constantly monitor the concentration on the chosen event so to avoid mind wandering (Tops et al., 2014). Once practitioners become familiar with the FAM technique and can easily sustain their attentional focus on an object for a considerable amount of time, they often progress to OMM. During OMM the focus of the meditation becomes the monitoring of awareness itself (Lutz et al., 2008; Vago and Silbersweig, 2012). In contrast to FAM, there is no object or event in the internal or external environment that the meditator has to focus on. The aim is rather to stay in the monitoring state, remaining attentive to any experience that might arise, without selecting, judging, or focusing on any particular object. To start, however, the meditator will focus on a chosen object, as in FAM, but will subsequently gradually reduce this focus, while emphasizing the activity of monitoring of awareness.

Loving-kindness meditation incorporates elements of both FAM and OMM (Vago and Silbersweig, 2012). Meditators focus on developing love and compassion first for themselves and then gradually extend this love to ever more “unlikeable” others (e.g., from self to a friend, to someone one does not know, to all living beings one dislikes). Any negative associations that might arise are supposed to be replaced by positive ones such as pro-social or empathic concern.

## MEDITATION TYPES, ATTENTIONAL SCOPE, AND ENDOGENOUS ATTENTION

Whereas some meditation techniques require the practitioners to focus their attention on only a certain object or event, other techniques allow any internal or external experiences or sensations to enter awareness. Different meditation techniques might therefore bias the practitioner to either a narrow or broad spotlight of attention. This distinction is thought to be most evident with regard to FAM and OMM. FAM induces a narrow attentional focus due to the highly concentrative nature of the meditation, whereas OMM induces a broader attentional focus by allowing and acknowledging any experiences that might arise during meditation.

In a seminal study, Slagter et al. (2007) investigated the effects of 3 months of intensive Vipassana meditation (an OMM-like meditation) training on the allocation of attention over time as indexed by the “attentional-blink” (AB) deficit, thought to result from competition between two target stimuli (T1 and T2) for limited attentional resources. After the training, because of the acquisition of a broader attentional scope, participants showed a smaller AB deficit as an indication of being able to distribute their brain-resource allocation to both T1 and T2. The reduced AB size was accompanied by a smaller T1-elicited P3b, a brain-potential thought to index attentional resource allocation.

A more recent study comparing meditators (trained in mindfulness-based stress-reduction) to non-meditators found that meditators show evidence of more accurate and efficient visual attention (Hodgins and Adair, 2010). Meditators monitored events more accurately in a concentration task and showed less interference from invalid cues in a visual selective attention task. Furthermore, meditators showed improved flexible visual attention by identifying a greater number of alternative perspectives in multiple perspectives images. Another study compared OMM and FAM meditators on a sustained attention task (Valentine and Sweet, 1999): OMM meditators outperformed FAM meditators when the target stimulus was unexpected. This might indicate that the OMM meditators had a wider attentional scope, even though the two meditator groups did not differ in performance when the stimulus was expected.

Electrophysiological evidence for meditation-induced improvements in attention comes from a recent study in which Vipassana meditators performed an auditory oddball task before and after meditation (in one session) and random thinking (in another session; Delgado-Pastor et al., 2013). The meditation session was composed by three parts. First, an initial part of self-regulation of attention focused on sensations from air entering and leaving the body at the nostrils. Second, a central part of focusing attention on sensations from all parts of the body while maintaining the non-reactivity and acceptance attitude. Last, a final brief part aimed on generating feelings of compassion and unconditional love to all living beings. Meditators showed greater P3b amplitudes to target tones after meditation than either before meditation or after the no-meditation session, an effect that is thought to reflect enhanced attentional engagement during the task.

Support for the assumption that FAM induces a narrow attentional focus comes from several studies that show that FAM increases sustained attention (Carter et al., 2005; Brefczynski-Lewis et al., 2007). Neuroimaging evidence by Hasenkamp et al. (2012) suggests that FAM is associated with increased activity in the right dorsolateral prefrontal cortex (dlPFC), which has been associated with “the repetitive selection of relevant representations or recurrent direction of attention to those items” (D’Esposito, 2007, p. 765 ). Thus, in the context of meditation experience, dlPFC might be involved in repeatedly redirecting or sustaining attention to the object of focus. It would be interesting to investigate whether this pattern of activation is unique to FAM or whether other kinds of meditation lead to similar increases in activity in the dlPFC. If the dlPFC is indeed involved in the repetitive redirection of attention to the same object of focus, then it should not be as active during OMM during which attention is more flexible and continuously shifted to different objects. Alternatively, however, if during OMM the meditator achieves a state of awareness where (only) awareness itself is the object of focus, the dlPFC might again play a role in maintaining this focus. Similarly, it would be interesting to examine how LKM modulates attentional processes and the activation of the dlPFC.

In a follow-up study, Hasenkamp and Barsalou (2012) found that, during rest, the right dlPFC connectivity to the right insula was improved in experienced meditators compared to novices. The authors suggest that improved connectivity with the right insula might reflect enhanced interoceptive attention to internal bodily states. In a support of this idea, a recent study reports that mindfulness training predicted greater activity in posterior insula regions during interoceptive attention to respiratory sensation (Farb et al., 2013). Various studies have shown theta activity to be increased during meditation, primarily OMM-like meditations (e.g., Baijal and Srinivasan, 2010; Cahn et al., 2010; Tsai et al., 2013; for review see Travis and Shear, 2010). This increase in theta activity, usually mid-frontal, has been suggested to be involved in sustaining internalized attention. As such, similar increases in theta activity would be expected for LKM during which attention is also internalized, but not during FAM where attention is explicitly focused on an external object, even though typically the object of meditation in FAM, at least for beginners, is the breath, which is internal.

Additionally, active mindfulness meditation (versus rest) was associated with increased functional connectivity between the dorsal attention network, the Default Mode Network and the right prefrontal cortex (Froeliger et al., 2012). Thus, meditation practice seems to enhance connectivity within and between attentional networks and a number of broadly distributed other brain regions subserving attention, self-referential, and emotional processes.

## MEDITATION TYPES AND CONFLICT MONITORING

A fundamental skill acquired through meditation is the ability to monitor the attentional focus in order to “redirect it” in the case of conflicting thoughts or external events. Not surprisingly, several studies have already shown improvements in conflict monitoring after meditation. Tang et al. (2007) investigated whether a training technique based on meditational practices called integrative body-mind training (IBMT; most similar to OMM) could improve performance on an attentional network task (ANT; Fan et al., 2002). The ANT was developed to keep track of three different measures, namely orientation, alerting, and conflict resolution. While IBMT had no effect on orienting and alerting scores, it did improve conflict resolution. In a similar study FAM and OMM were compared on an emotional variant of the ANT. Both types of meditation improved conflict resolution compared to a relaxation control group (Ainsworth et al., 2013). Surprisingly, there was no difference between the two meditation types, even though, mindfulness disposition at baseline (i.e., trait mindfulness) was also associated with improved conflict resolution.

Further evidence for improvements in conflict monitoring come from a study investigating the effect of 6-week long FAM trainig (versus relaxation training and a waiting-list group) on a discrimination task intended to investigate the relationship between attentional load and emotional processing (Menezes et al., 2013). Participants had to respond to whether or not the orientation of two lines presented to either side of an emotionally distracting picture was the same. Importantly, those who underwent a meditation or relaxation training commited fewer errors than the waiting list control group. Furthermore, error rates were lowest in the meditation group, higest in the waiting list group, while the relaxation group scored in between. With regard to emotional regulation meditators showed less emotional interference than the other two groups when attentional load was low, and only meditators showed a relationship between the amount of weekly practice and reductions in emotional interference.

In a study of Xue et al. (2011), meditation-naïve participants were randomly assigned to either an 11 h IBMT course or a relaxation training. Compared to the relaxation training, the IBMT group showed higher network efficiency and degree of connectivity of the anterior cingulate cortex (ACC). As the ACC is involved in processes such as self-regulation, detecting interference and errors, and overcoming impasses (e.g., Botvinick et al., 2004), improvements in ACC functioning might well be the neural mechanism by which IBMT improves conflict resolution. In an interesting study of Hasenkamp et al. (2012), experienced meditators engaged in FAM inside an fMRI scanner and pushed a button whenever they started to mind-wander. The moment of awareness of mind-wandering was associated with increased activity in the dorsal ACC. Thus, as the mind starts to wander during meditation, the ACC might detect this “error” and feed it back to executive control networks (Botvinick et al., 1999; Carter and van Veen, 2007), so that attention can be refocused. Various other studies have also shown improvements in ACC functioning after meditation (Lazar et al., 2000; Baerentsen et al., 2001; Tang et al., 2009, 2010). Hölzel et al. (2007) compared experienced and novice meditators during a concentrative meditation (akin to FAM) and found that the experienced meditators showed greater activity in the rostral ACC during meditation than the novices, even though the two groups did not differ on an arithmetic control task. Similar results were obtained in another study comparing novices and experienced meditators (Baron Short et al., 2007) by showing more activity in the ACC during FAM compared to a control task. The activity in the ACC was more consistent and sustained for experienced meditators. Related to that, Buddhist monks exhibited more activity in the ACC during FAM than during OMM (Manna et al., 2010). This suggests that the effects of meditation on the ACC and conflict monitoring do not seem to be limited to temporary state effects but carry over into daily life as a more stable “trait.” Future large scale longitudinal studies should to be conducted to address this issue and to disentangle short-term and long-term effects on conflict monitoring.

Improved conflict monitoring does not necessarily entail increased brain activity. Kozasa et al. (2012) compared meditators and non-meditators on a Stroop task in which semantic associations of words have to be suppressed to retrieve the color of the word. While behavioral performance was not significantly different for the two groups, compared to meditators, the non-meditators showed more activity in brain regions related to attention and motor control during incongruent trials. Given that the aim of many meditation techniques is to monitor the automatic arise of distractible sensations, such skill may become effortless by repeated meditation, therefore leading to less brain activity during the Stroop task. LKM has been shown to improve conflict resolution, as well, when LKM and a control group were compared on a Stroop task. The LKM group was faster in responding to both congruent and incongruent trials, and the difference between congruent and incongruent trials (the congruency effect) was smaller as well (Hunsinger et al., 2013). As LKM incorporates elements of both FAM and OMM, it would be interesting to investigate how the effect size associated with LKM may be positioned in between FAM and OMM.

Recently, meditators and non-meditators were compared with regard to measures of cortical silent period and short intra cortical inhibition over the motor cortex before and after a 60 min long meditation (for the meditators) or cartoon (for the non-meditators), respectively, measuring GABA_B_ receptor-mediated inhibitory neurotransmission and GABA_A_ receptor-mediated inhibitory neurotransmission (Guglietti et al., 2013). Given that deficits related to cortical silent periods in the motor cortex had been previously associated with psychiatric illness and emotional deregulation, the activity over the motor cortex was measured. No differences were found between meditators and non-meditators before the meditation/cartoon. However, after meditation there was a significant increase in GABA_B_ activity in the meditator group. The authors suggest that “improved cortical inhibition of the motor cortex, through meditation, helps reduce perceptions of environmental threat and negative affect through top down modulation of excitatory neural activity” (Guglietti et al., 2013, p. 400). Future research might investigate whether similar GABA related mechanisms underlie the suppression of distracting stimuli during meditation and how different types of meditation might have distinguishable effects on these processes.

## MEDITATION TYPES AND CREATIVITY

The scientific evidence regarding the connection between meditation and creativity is inconsistent. While some studies support a strong positive impact of meditation practice on creativity (Orme-Johnson and Granieri, 1977; Orme-Johnson et al., 1977), others found only a weak association or no effect at all (Cowger, 1974; Domino, 1977). Recently, Zabelina et al. (2011) found that a short-term effect of mindfulness manipulation (basically OMM) facilitated creative elaboration at high levels of neuroticism. As pointed out by Colzato et al. (2012), these inconsistencies might reflect a failure to distinguish between different and dissociable processes underlying creativity, such as convergent and divergent thinking (Guilford, 1950). Accordingly, Colzato et al. (2012) compared the impact of FAM and OMM on convergent thinking (a process of identifying one “correct” answer to a well-defined problem) and divergent thinking (a process aiming at generating many new ideas) in meditation practitioners. Indeed, the two types of meditation affected the two types of thinking in opposite ways: while convergent thinking tended to improve after FAM, divergent thinking was significantly enhanced after OMM. Colzato et al. (2012) suggest that FAM and OMM induce two different, to some degree opposite cognitive-control states that support state-compatible thinking styles, such as convergent and divergent thinking, respectively. In contrast to convergent thinking, divergent thinking benefits from a control state that promotes quick “jumps” from one thought to another by reducing the top-down control of cognitive processing—as achieved by OMM.

## CONCLUSION

Research on meditation is still in its infancy but our understanding of the underlying functional and neural mechanisms is steadily increasing. However, a serious shortcoming in the current literature is the lack of studies that systematically distinguish between and compare different kinds of meditation on various cognitive, affective or executive control tasks—a criticism that applies to neuroscientific studies in particular. Further progress will require a better understanding of the functional aims of particular meditation techniques and their strategies to achieve them. It will also be important to more systematically assess short- and long-term effects of meditation, as well as the (not yet understood) impact of meditation experience (as present in practitioners but not novices). For instance, several approaches (like Buddhism) favor a particular sequence of acquiring meditation skills (from FAM to OMM) but evidence that this sequence actually matters is lacking. Moreover, the neural mechanisms underlying meditation effects are not well understood. It might be interesting that the three main research topics we have covered in the present review (attentional control, performance monitoring, and creativity or thinking style) imply the operation of extended neural networks, which might suggest that meditation operates on neural communication, perhaps by impacting neurotransmitter systems. Finally, it may be interesting to consider individual differences more systematically. If meditation really affects interactions between functional and neural networks, it makes sense to assume that the net effect of meditation of performance depends on the pre-experimental performance level of the individual—be it in terms of compensation (so that worse performers benefit more) or predisposition (so that some are more sensitive to meditation interventions).

## Conflict of Interest Statement

The authors declare that the research was conducted in the absence of any commercial or financial relationships that could be construed as a potential conflict of interest.

## Footnotes

1 It is important to note that even though this mini review is based on the theoretical framework of distinguishing FAM and OMM, another one includes the distinction between concentrative meditations, practices that regulate or control attention/awareness, and meditation practices which instead do not explicitly target attentional/effortful control (Chiesa and Malinowski, 2011; see also Chiesa, 2012 for a recent review on the difficulty of defining Mindfulness). Moreover, Travis and Shear (2010) have pointed out a third meditation category besides FAM and OMM: the *automatic self-transcending* which trascends FAM and OMM through the absence of both (a) focus and (b) individual control or effort.
